# Characteristics and Risk Factors of Falls Within a Residential Environment: A Follow-Up Study of Community-Dwelling Japanese Adults Aged 75 Years and Older Requiring Long-Term Care

**DOI:** 10.7759/cureus.80924

**Published:** 2025-03-20

**Authors:** Ryuichi Sawa, Buichi Tanaka, Junshiro Yamamoto, Minoru Yamada

**Affiliations:** 1 Faculty of Health Science, Juntendo University, Bunkyo, JPN; 2 Geriatrics, Hakuyu Co. Ltd., Nagano, JPN; 3 Geriatrics, EveRehab, Inc., Kyoto, JPN; 4 Graduate School of Health Science, University of Tsukuba, Tsukuba, JPN

**Keywords:** accidental falls, circumstances, environmental hazards, frail older adults, rural japan

## Abstract

Introduction

Understanding the characteristics of falls is crucial for effectively managing them in later life. Environmental hazards play a significant role in fall incidents with age-related life space constriction. This study explored the characteristics of falls within the home and the impact of environmental fall hazards on fall incidents among community-dwelling Japanese adults aged ≥75 years with long-term care needs.

Methods

This follow-up study, conducted in an outpatient day long-term care facility, enrolled 96 community-dwelling adults aged ≥75 years with long-term care needs. The occurrence and circumstances of falls within the home were recorded biweekly through face-to-face interviews during the follow-up period. Falls were categorized as “any fall” and “serious falls.” Descriptive analyses were used to summarize fall characteristics. Univariate and multivariate Cox proportional hazards regression models explored the impact of environmental hazards on the time to first fall and serious falls.

Results

During the follow-up period, 22 participants (23.0%) had at least one fall. The most frequent fall locations were shared rooms and outdoor spaces, each accounting for approximately 30% of all reported falls. Walking was the most common activity at the time of falls (32.6%), and participants frequently attributed falls to loss of balance (46.5%). A higher number of environmental hazards significantly elevated the risk of any fall (HR: 1.15, 95% CI: 1.06-1.25, *p* < 0.05). For serious falls, environmental hazards (HR: 1.11, 95% CI: 1.00-1.23, *p* < 0.05) were significantly associated with the occurrence of serious falls within the home.

Conclusions

We clarified the characteristics of falls in Japanese residential environment. Assessment of environmental hazards is essential for fall prevention and management, especially within residential environments, to allow older adults to continue to live in their own homes.

## Introduction

The global demographic structure is shifting with an increasing trend toward population aging. A country is classified as an “aging society” when more than 7% of its population is ≥65 years, an “aged society” at over 14%, and a “super-aged society” at over 21% [1]. Japan reached the status of a super-aged society in 2007, and the percentage continues to grow. The current characteristic of population aging in Japan is defined by the demographic structure of the population aged ≥65 years. In 2023, the percentage of the population aged ≥75 years surpassed that of the population aged 65-74 years (16.1% vs. 13.0%) and is expected to increase further. This demographic shift has significant implications for care needs, as many of the population aged ≥75 years continue to live in their communities while receiving support through the Japanese long-term care insurance (LTCI) system.

Given the global demographic shift toward an aging population, falls among older adults represent one of the most critical public health concerns [2]. Approximately one-third of older adults experience at least one fall annually [3]. Falls adversely affect functional independence and quality of life [4-6]. Both injurious and multiple falls are associated with an increased risk of hospitalization and nursing home admission [7,8], placing a substantial burden on public healthcare systems [5,9,10]. The risk of falls among older adults is determined by the complex interaction between individual and environmental factors in daily life [11]. Individual factors such as physical and cognitive functions gradually decline with age [12], and consequently, older adults’ life space mobility becomes restricted within homes [13]; therefore, the degree of the interaction is likely to differ between age groups, specifically between those aged 65-74 years and those aged ≥75 years. Many studies have grouped adults aged ≥65 years together; however, the classification may overlook the differences in the characteristics of falls between age groups. In light of the changing demographic structure in Japan, we believe that clarifying the characteristics of falls among older adults aged ≥75 years is of significant importance.

We hypothesize that environmental hazards play a relatively significant role in fall occurrence within Japanese residential environments compared with other risk factors. There are many environmental fall hazards within homes such as clutter, loose rugs, and inadequate lighting [14]. Additionally, Japanese housing structures and cultural characteristics may have the potential for increasing exposure to hazardous environments in daily life. Traditional Japanese housing incorporates features such as multiple door sills within homes. Japanese residential practices include changing footwear at the house entrance, where outdoor shoes are exchanged for indoor slippers or removed entirely. For activities in residential outdoor spaces such as gardens, Japanese residents typically wear easily removable footwear such as sandals. Furthermore, the cultural practice of placing items directly on the floor is common in Japanese households. These distinctive cultural characteristics may influence the circumstances of falls within the home and how individual and environmental factors interact to cause falls in daily life. Given that home fall-hazard interventions have been reported as effective for high-risk older adults [15], there is potential for even greater intervention efficacy in typical Japanese residential environments.

Our previous cross-sectional study examined environmental hazards within homes and demonstrated their association with daily experiences of trips and slips, but not with falls, in community-dwelling Japanese older adults aged ≥75 years requiring long-term care [16]. Cross-sectional studies relying on retrospective fall data are susceptible to recall bias [17,18], which may be particularly pronounced among older adults, especially those aged ≥75 years, because of age-related memory impairment [19]. This study aimed to explore the characteristics of falls within the home and the impact of environmental fall hazards on fall incidents among community-dwelling Japanese adults aged ≥75 years with long-term care needs.

## Materials and methods

Study design and participants

This study was conducted as part of the project for developing a self-assessment tool for fall hazards in residential environments. The project aims to reduce fall hazards in residential environments in order to achieve aging-in-place among older adults, even those requiring long-term care needs. To enroll community-dwelling older adults with long-term care needs, the inclusion criteria were: individuals who used an outpatient day long-term care facility under the Japanese LTCI system were aged ≥75 years, had care certification of any level, and were able to walk independently within the home. Those who were diagnosed with dementia, had cognitive impairment (defined as a score of <24 on the Mini-Mental State Examination [20]), were living in residential facilities (e.g., nursing homes or long-term care facilities), or had incomplete data were excluded. Cross-sectional findings from the baseline survey were reported previously [16], and this study represents a follow-up analysis of prospectively collected data. All participants provided written informed consent in accordance with the Declaration of Helsinki. Ethical approval for this study was granted by the Ethics Committee of the Juntendo University Faculty of Health Science (approval number 23-037).

Baseline measurements

Demographic characteristics were recorded through face-to-face interviews, including age, sex, cohabitation status, the level of care certification in the Japanese LTCI system, the number of comorbidities (hypertension, diabetes mellitus, stroke, osteoporosis, heart disease, respiratory disease, rheumatoid arthritis, hip osteoarthritis, and knee osteoarthritis), and the number of medications. Age was categorized into two groups: 75-84 years old and ≥85 years old [21]. Participants were classified as either “living alone” or “living together” on the basis of the information obtained on cohabitation status. Polypharmacy was defined as taking more than five medications per day [22].

Physical performance was assessed using three standardized clinical measurements: the timed up and go test (TUG), handgrip strength, and gait speed. For the TUG, participants performed a sequence of functional movements: rising from a chair, walking 3 meters at their usual pace, turning 180 degrees, returning to the chair, and sitting down. TUG scores were recorded in seconds, with lower values indicating better functional mobility and dynamic balance capability. Handgrip strength was measured using a Smedley-type handheld dynamometer (GRIP-D; Takei Ltd., Niigata, Japan). Gait speed was calculated on the basis of the time taken for participants to walk through the central (1 meter) part of a 5-meter walkway at their usual speed. Global cognitive function was assessed using the Mini-Mental State Examination [20].

Environmental hazard assessment was conducted using a research-focused instrument designed for the Japanese housing structure [16]. This tool evaluated 46 potential hazards across 13 environmental categories within the home: (1) approach; (2) entrance; (3) living room; (4) kitchen; (5) bedroom; (6) toilet; (7) lavatory; (8) bathroom; (9) balcony; (10) garden; (11) hallways; (12) stairs; and (13) light and indoor footwear. A score of “1” was assigned for a given item if the hazard was present, with the total score ranging from 0 to 46 (the sum score for the items on which the participants answered “yes”). A higher score indicated greater environmental hazard exposure within the residential setting. Our previous study revealed that the score of the tool was significantly related to near-falls (OR: 1.11, 95% CI: 1.02-1.21, *p* < 0.05), which demonstrates its concurrent validity [16].

Falls

A fall was defined as “an unexpected event in which the person comes to rest on the ground, floor, or a lower level.” After reviewing this standardized definition with the participants, we collected both retrospective and prospective fall data. The baseline assessment included fall history in the previous six months, fall frequency, and fall location within the home. The prospective monitoring protocol involved biweekly structured interviews conducted by trained care staff, documenting fall occurrence, spatial location within the home (approach, entrance, living room, kitchen, bedroom, toilet, lavatory, bathroom, balcony, garden, hallways, stairs, or others), activities at the time of the fall (walking, weight shifting, turning, putting on/taking off footwear, or using the stairs), causes of fall (tripping, slipping, or loss of balance), and associated injuries. One of our researchers was directly involved in the staff training, instructing interviewers to share the definition of a fall with participants at the beginning of each interview. Care staff could consult with this researcher whenever they encountered cases that were difficult to assess. Participants were censored if they were absent for more than one month or discontinued day care services. The follow-up period was from May to October 2021 (mean follow-up duration: 24.6 weeks; maximum: 26.0 weeks).

Statistical analysis

Descriptive statistics for continuous variables were expressed as medians with IQRs, whereas categorical variables were reported as absolute numbers with percentages. Fall incidence was calculated as the number of falls per person-year, with both the cumulative fall count and the proportion of fallers during the follow-up period being documented.

Univariate and multivariate Cox proportional hazard models were used to estimate the HRs and 95% CIs of variables potentially influencing the time to the first fall and serious falls. Serious falls were defined as either injurious falls or multiple falls, which are more likely to seriously impact a person’s life [23]. The time to serious falls was defined as the time taken to experience either an injurious fall or a second fall. The number of environmental hazards was set as the primary explanatory variable. Age, female sex, the number of comorbidities, polypharmacy, fall history in the past six months, and TUG were included as covariates [24]. The number of environmental hazards, number of comorbidities, and TUG were analyzed as continuous variables, and the others as categorical variables. All analyses were performed using IBM SPSS Statistics for Windows, Version 28.0 (Released 2021; IBM Corp., Armonk, NY, USA). The significance level for all analyses was set at* p* < 0.05.

## Results

Of 97 older adults enrolled at baseline, 96 participated in this study. One enrollee at baseline had discontinued using the outpatient day long-term care facility before the start of the follow-up period. The demographic characteristics at the beginning of the follow-up period are listed in Table 1. The mean age of the participants was 86.5 ± 4.4 years (median = 87 years; range: 75-100 years), and 72 participants (75.0%) were women. The proportion of polypharmacy was 55.2%, and the number of environmental hazards ranged from 3 to 30 (median = 13). There were 20 (20.8%) participants who reported at least one fall in and around the home in the past six months.

**Table 1:** Baseline characteristics of the study participants (n = 96) All continuous variables are presented as median values with IQR and all categorical variables as numbers with percentages. TUG, timed up and go test

Characteristics	Median (IQR)/no. (%)
Age, year	87 (84, 89)
Female sex	72 (75.0)
Care-needs certification	
Support level 1	39 (40.6)
Support level 2	38 (39.6)
Care level 1	13 (13.5)
Care level 2	5 (5.2)
Care level 3	1 (1.0)
Living alone	42 (43.8)
Polypharmacy	53 (55.2)
Number of comorbidities, number	2 (1, 3)
Comorbidities	
Hypertension	56 (58.3)
Diabetes	12 (12.5)
Stroke	9 (9.4)
Osteoporosis	30 (31.3)
Heart diseases	25 (26.0)
Respiratory diseases	9 (9.4)
Rheumatoid arthritis	3 (3.1)
Hip osteoarthritis	10 (10.4)
Knee osteoarthritis	29 (30.2)
Physical performance	
TUG, seconds	8.7 (7.3, 10.5)
Gait speed, m/s	1.2 (1.0, 1.4)
Handgrip strength, kg	17.9 (14.8, 22.1)
Fall history in the past six months	20 (20.8)
Mini-Mental State Examination, score	28 (26, 29)
Number of environmental hazards, number	13 (10, 19)

The fall incidence rate was 0.9 falls per person-year. During the follow-up period, 22 participants (22.9%) experienced 43 falls, with eight (36.4%) reporting multiple falls. Nine fallers (40.9%) sustained injuries. Twelve participants (54.5%) met the criteria for serious falls. Table 2 shows the spatial distribution of falls within the home environment. Falls occurred in shared rooms (n = 14, 32.6%), non-shared rooms (n = 2, 4.7%), internal circulation spaces (n = 6, 14.0%), outdoor residential areas (n = 15, 34.9%), and other/unspecified locations (n = 4, 14.0%). The most frequent activities at the time of the fall were walking (n = 14, 32.6%) and weight shifting (n = 7, 16.3%). Loss of balance was the predominant cause, accounting for 20 falls (46.5%).

**Table 2:** Circumstances of falls including locations, activities, and self-reported causes of falls occurred within homes during the follow-up period (n = 43) Values are presented as n (% of total 43 fall events) [% excluding missing values]. ^†^ There were two falls with missing information on specific locations. ^‡^ There were 14 falls with missing information on specific activities at the time of the falls. ^§^ There were four falls with missing information on self-reported causes of falls.

Circumstances of falls	
Locations^†^	
Shared spaces	14 (32.6) [34.1]
Living or dining room	8 (18.6) [19.5]
Kitchen	2 (4.7) [4.9]
Bedroom	4 (9.3) [9.8]
Non-shared spaces	2 (4.7) [4.9]
Entrance	0 (0.0) [0.0]
Toilet	2 (4.7) [4.9]
Lavatory	0 (0.0) [0.0]
Bathroom	0 (0.0) [0.0]
Balcony	0 (0.0) [0.0]
Internal circulation spaces	6 (14.0) [14.6]
Hallways	5 (11.6) [12.2]
Stairs	1 (2.3) [2.4]
Outside residential areas	15 (34.9) [36.6]
Approach	3 (7.0) [7.3]
Garden	12 (27.9) [29.3]
Other/unspecified locations	4 (9.3) [9.8]
Activity^‡^	
Walking	14 (32.6) [48.3]
Weight shifting	7 (16.3) [24.1]
Turning	3 (7.0) [10.3]
Putting on/off footwear	3 (7.0) [10.3]
Stepping stairs	2 (4.7) [6.9]
Self-reported cause of falls^§^	
Loss of balance	20 (46.5) [51.3]
Tripping	11 (25.6) [28.2]
Slipping	8 (18.6) [20.5]

Cox proportional hazards analysis revealed that the number of environmental hazards significantly increased the risk of any fall within the home (HR: 1.15, 95% CI: 1.06-1.25). For serious falls, environmental hazards (HR: 1.11, 95% CI: 1.00-1.23) were significantly associated with the occurrence of serious falls within the home (Table 3).

**Table 3:** HRs for any fall incident and for serious fall incident ^†^ Any fall was defined as a single fall regardless of injuries. The time to any fall was defined as the time taken to experience the first fall. ^‡ ^Serious falls were defined as either an injurious fall or multiple falls. The time to serious falls was defined as the time taken to experience either an injurious fall or a second fall. For detailed baseline characteristics of participants, see Table 1. TUG, timed up and go test

Variable	Any fall^†^		Serious fall^‡^	
	Univariate (HR [95% CI])	Multivariate (HR [95% CI])	Univariate (HR [95% CI])	Multivariate (HR [95% CI])
Age	1.18 (0.46, 3.02)	1.04 (0.40, 2.75)	2.25 (0.49, 10.30)	2.24 (0.46, 10.83)
Female sex	1.19 (0.44, 3.22)	1.01 (0.34, 3.02)	1.04 (0.28, 3.84)	1.06 (0.25, 4.54)
Number of comorbidities	1.09 (0.77, 1.53)	1.19 (0.83, 1.72)	1.51 (0.98, 2.34)	1.46 (0.91, 2.35)
Polypharmacy	0.57 (0.24, 1.32)	0.42 (0.17, 1.05)	1.19 (0.38, 3.75)	0.64 (0.18, 2.33)
Fall history in the past six months	1.73 (0.71, 4.24)	2.37 (0.86, 6.53)	0.72 (0.16, 3.30)	1.06 (0.21, 5.28)
TUG	1.01 (0.91, 1.11)	0.99 (0.88, 1.11)	1.06 (0.96, 1.16)	1.04 (0.94, 1.16)
Number of environmental hazards	1.11 (1.03, 1.19)	1.15 (1.06, 1.25)	1.12 (1.01, 1.23)	1.11 (1.00, 1.23)

## Discussion

This follow-up study characterized fall events within the home among Japanese adults aged ≥75 years requiring long-term care, examining fall locations, activities, and self-reported causes. Common fall locations within the residential environment were the living or dining room and garden. Walking was the most common activity at the time of falls, and participants frequently attributed falls to loss of balance. A greater number of environmental hazards within the residential environment was significantly associated with an increased probability of fall occurrence during the follow-up period.

Consistent with previous studies, falls frequently occurred in shared spaces, particularly the living room and bedroom [21]. Bathrooms were identified as high-risk locations for falls within residential environments, with some studies on fall-related injuries and hospitalizations documenting a high prevalence of bathroom-related incidents [25]. However, no bathroom-related falls were reported in this study, possibly because we included both injurious and non-injurious falls. This finding aligns with a recent study reporting that only 51 out of 1,829 falls (2.8%) occurred in bathrooms [26]. Given the known tendency for underreporting of falls among older adults [27], the actual distribution of fall locations within the home may be misrepresented in large-scale studies, including those using national surveillance data [25].

Walking was identified as the most common activity at the time of falls in this study, in agreement with previous findings [26]. Walking in loose footwear (e.g., bare feet, less “fixed” footwear, or slippers) increased joint angle variability and decreased minimum toe clearance [28,29], thereby elevating fall risk. It is a traditional Japanese residential custom to change footwear at the house entrance to indoor slippers or to remove footwear entirely. Gardens, the most common location for falls in our study, are outdoor spaces within residential properties where residents frequently perform household activities such as gardening and hanging laundry. For these activities, Japanese residents typically choose easily removable footwear, such as sandals, to facilitate the frequent transitions between indoor and outdoor spaces. Garden activities often involve turning and weight-shifting movements that challenge balance. We suggested that the combination of these balance-challenging movements while wearing loose footwear may contribute to the high prevalence of garden falls. Given the cultural uniqueness of footwear practices in Japanese homes, the type of footwear used in and around the home should be considered an important environmental factor in fall risk assessments in Japan.

Our findings confirmed the high prevalence of environmental hazards within the home, consistent with previous research [21]. Falls result from the interaction between individual and environmental factors [11]. Individual risk factors include sociodemographic, health-related, and behavioral characteristics, whereas environmental factors encompass all external attributes such as neighborhood characteristics and housing structures. The traditional Japanese lifestyle involves spending considerable time sitting on the floor, with various items such as clothing, newspapers, books, and cushions commonly placed on floor surfaces. This cultural practice necessitates stepping over floor-level objects during daily activities, potentially increasing fall risk while walking. Furthermore, older adults typically spend more time in their residential environment [13], thereby increasing their exposure to these environmental hazards. Our findings demonstrated that the number of environmental hazards within the home, and not physical functioning, significantly predicted the occurrence of falls in and around the home during the follow-up period. In examining the interaction between individual and environmental factors, our findings suggest that environmental factors may have a stronger influence on fall occurrence within the home in the population.

Our findings demonstrated that the risk of serious falls, defined as injurious or multiple fall events [23], in residential environments increased with a greater number of environmental hazards. These serious falls have been associated with adverse outcomes such as institutionalization and hospitalization [7,8], supporting the recommendation for their separate analysis in fall-related research [24]. Additionally, indoor falls serve as an indicator of functional decline among older adults, even in the absence of fracture [30]. Several studies have highlighted differences in characteristics between older adults who experience outdoor falls versus those who fall indoors [31,32]. Characteristics of outdoor fallers included younger age, higher physical activity, and better health status, whereas indoor fallers were characterized by advanced age, frailty, and poor health status. Our findings align with these characteristics and suggest that exposure to environmental hazards contributes to the occurrence of serious falls within residential environments. Home modifications are a way to mitigate fall risk and improve daily activities in older adults [33,34]. Given that the effects of exercise intervention on fall prevention in people aged ≥75 years are limited [35], home modifications are essential for older adults with age-related functional limitations to achieve safe and independent living in their own homes.

Our previous study using a cross-sectional design showed that the number of environmental hazards was not associated with past fall experience, but was associated with daily trips and slips [16]. In contrast, the current study showed a significant association between the number of environmental hazards and falls. One of the challenges facing studies on falls, particularly in older adults, is recall bias. A systematic review to explore the appropriate interval to recall falls is recommended, following up at least every month [18]. The participants in the current study were cognitively intact and were followed up biweekly to gather information on falls; therefore, our findings reinforce the evidence regarding the association between environmental hazards and falls in older adults. This short follow-up interval minimizes the influence of recall bias and was a major strength of the current study. However, this study also has several limitations. The sample size was relatively small. Another limitation was that we recruited only older adults who used an outpatient day long-term care facility, a service provided under LTCI in Japan, with a convenience sampling method. The participants all lived in their own communities with some support under the LTCI system; therefore, further studies with more diverse samples including those without formal care services should be conducted to improve the generalizability of the findings.

## Conclusions

This study presented descriptive data on the characteristics of falls within homes and provided preliminary evidence that the number of environmental hazards is a risk factor for future falls within the home among community-dwelling Japanese adults aged ≥75 years with long-term care needs. Falls occurred not only in indoor spaces but also in outdoor spaces inside the property. Gardens may be locations with a higher risk of falls in residential environments, and further studies should explore the specific characteristics of falls in these areas. This population desires “aging-in-place,” i.e., remaining in their familiar environment for as long as possible, and our findings emphasize the importance of assessing environmental hazards for fall prevention and management to allow older adults to continue to live in their own homes even with necessary day care support.
